# Rosette-Disrupting Effect of an Anti-Plasmodial Compound for the Potential Treatment of *Plasmodium falciparum* Malaria Complications

**DOI:** 10.1038/srep29317

**Published:** 2016-07-11

**Authors:** Jun-Hong Ch’ng, Kirsten Moll, Maria del Pilar Quintana, Sherwin Chun Leung Chan, Ellen Masters, Ernest Moles, Jianping Liu, Anders B. Eriksson, Mats Wahlgren

**Affiliations:** 1 Department of Microbiology, Tumor and Cell Biology (MTC), Karolinska Institutet, Stockholm, Sweden; 2 Department of Microbiology, National University of Singapore, Singapore; 3 Escuela de Medicina y Ciencias de la Salud, Facultad de Ciencias Naturales y Matemáticas, Universidad del Rosario, Bogotá, Colombia; 4 Life Sciences Department, Imperial College London, London, England; 5 Nanomalaria Group, Institute for Bioengineering of Catalonia (IBEC), Barcelona, Spain; 6 Barcelona Institute for Global Health (ISGlobal, Hospital Clínic-Universitat de Barcelona), Barcelona, Spain; 7 Nanoscience and Nanotechnology Institute (IN2UB), University of Barcelona, Barcelona, Spain; 8 Karolinska High Throughput Centre (KHTC), Karolinska Institutet, Stockholm, Sweden

## Abstract

The spread of artemisinin-resistant parasites could lead to higher incidence of patients with malaria complications. However, there are no current treatments that directly dislodge sequestered parasites from the microvasculature. We show that four common antiplasmodial drugs do not disperse rosettes (erythrocyte clusters formed by malaria parasites) and therefore develop a cell-based high-throughput assay to identify potential rosette-disrupting compounds. A pilot screen of 2693 compounds identified Malaria Box compound MMV006764 as a potential candidate. Although it reduced rosetting by a modest 20%, MMV006764 was validated to be similarly effective against both blood group O and A rosettes of three laboratory parasite lines. Coupled with its antiplasmodial activity and drug-likeness, MMV006764 represents the first small-molecule compound that disrupts rosetting and could potentially be used in a resource-limited setting to treat patients deteriorating rapidly from malaria complications. Such dual-action drugs that simultaneously restore microcirculation and reduce parasite load could significantly reduce malaria morbidity and mortality.

Cerebral malaria (CM) still occurs in about 1% of P*lasmodium falciparum* infections, mostly among children^1^. Even with the recommended intravenous artesunate, treatment failure is still between 15–20%^2^. Additionally, 10–20% of survivors experience long-term neurological sequelae making CM a leading cause of childhood neuro-disability in the Sub-Saharan region^2^. Complications like CM occur when mature blood-stage parasites (trophozoites and schizonts) sequester in the microvasculature, inadvertently leading to vascular occlusion, tissue hypoxia, inflammation and multi-organ failure^1^.

Sequestration occurs through the direct cytoadhesion of the infected erythrocyte (iRBC) to the vascular endothelium, or through iRBC binding to uninfected erythrocytes (uRBC), leading to the formation of cell clusters called rosettes^2^. Rosetting results in microvasculature obstruction^3^ and has been clearly associated with severe malaria^4^. Both mechanisms are mediated by parasite proteins that are exported to the host erythrocyte surface belonging to the *P. falciparum* Erythrocyte Membrane Protein 1 (PfEMP1) family^5^, repetitive interspersed family (RIFINs)^6^ and subtelomeric variable open reading frame (STEVORs)^7^ gene families. Rosetting has also been observed in other *Plasmodia* species and may contribute to pathology in non-falciparum malaria as well^8^,^9^.

In this paper, we show that antiplasmodial chemotherapy does not affect rosetting and that blood group A rosettes are particularly resistant to heparin-induced dispersion. We then developed a cell-based high-throughput screen and identified an antiplasmodial Malaria Box compound with rosette-disrupting activity.

## Results

### Stage-specific staining and multiplet discrimination

**Figure 1:**
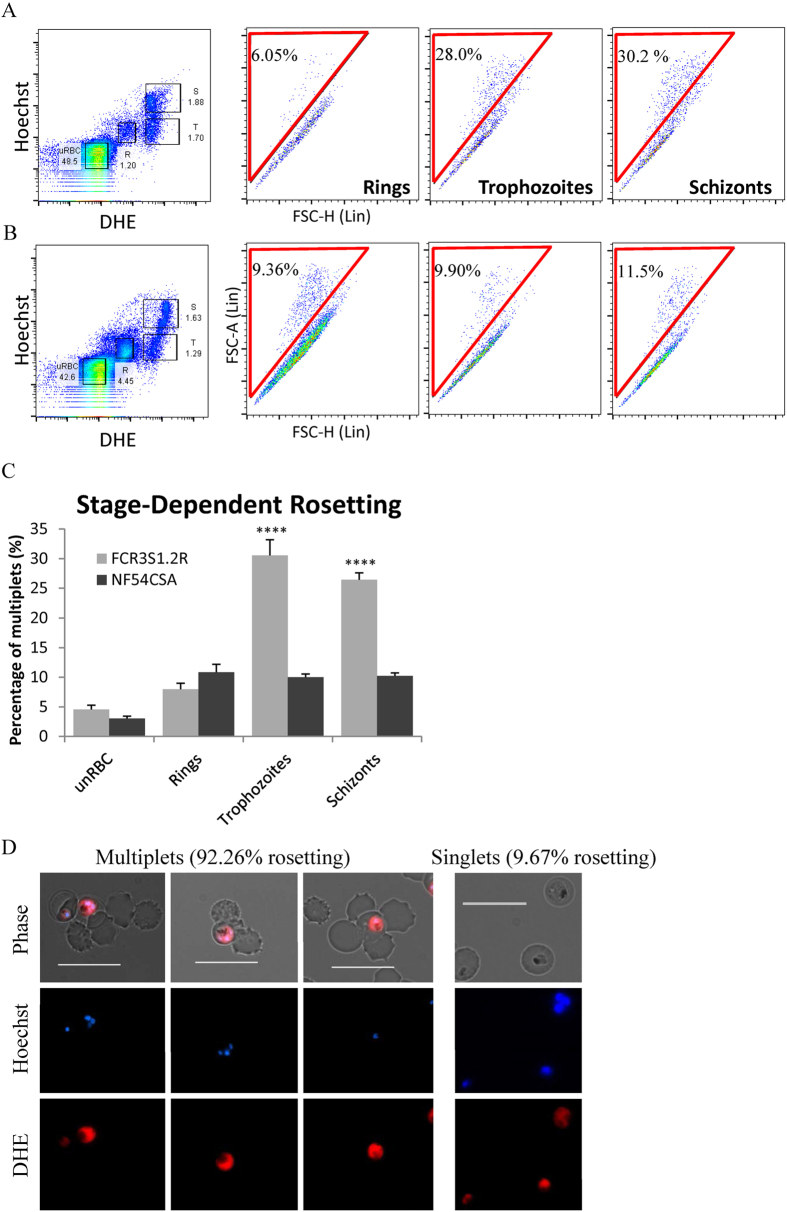
Strain- and stage-dependent presentation of multiplets. Cultures of (**A**) rosetting FCR3S1.2R and (**B**) non-rosetting NF54CSA parasites were co-stained with Hoechst and Dihydroethidium (DHE) to differentiate uninfected erythrocytes (uRBC) from ring- (R), trophozoite- (T) and schizont-infected (S) erythrocytes. The percentage of multiplets of each parasite stage were determined by gating for events that did not have a proportional forward scatter area (FSC-A) to forward scatter height (FSC-H) ratio. (**C**) The percentage of multiplets of each parasite is shown at different stages of parasite development (****P < 0.0001, N = 5). (**D**) Representative micrographs of multiplet-sorted (left three panels) and singlet-sorted (right-most panel) late-stage FCR3S1.2R parasites, with corresponding rosetting rate determined by microscopic enumeration of at least 300 parasites.

To gate for iRBCs in a stage-selective manner, we used a combination of Hoechst and DHE which has been well characterized^10^ and allows the discrimination of uninfected, ring-, trophozoite- and schizont-infected erythrocytes (Fig. 1A,B). The percentage of multiplets was measured by gating for events that fell above the 45° plane in the forward scatter area versus forward scatter height (FSCA-FSCH) plot.

In NF54CSA cultures, mature and immature parasites showed approximately 10% multiplets (Fig. 1C). In comparison, rosetting FCR3S1.2R late-stages had approximately 30% multiplets, a marked increase compared to ring-infected erythrocytes that have yet to express/export rosette-forming proteins (*P* < 0.0001, Fig. 1C).

These multiplets from late-stage FCR3S1.2R parasites were sorted for and rosettes of various sizes were collected (Fig. 1D left panels). These were imaged by fluorescence microscopy and the rosetting rate quantified to be in excess of 90%. Some unattached uRBCs were also observed, but these are likely to have been cells dislodged from rosettes since only Hoechst- and DHE-positive events were collected. In comparison, sorting for singlets revealed predominantly single cells (rosetting rate below 10%, Fig. 1D right-most panel).

Multiplet determination can also be performed by comparing FSC-A to FSC-Width, side-scatter (SSC)-Area to SSC-Height or SSC-Width to SSC-Area. While all plots could differentiate between rosetting and non-rosetting cells (data not shown), FSCA-FSCH was chosen since it was simplest to gate. We also validated the assay on BD LSRII, CyAn ADP Analyzer (Beckman-Coulter) and BD FACSVantage SE and observed that the assay was reproducible across cytometers (data not shown).

### Correlation between rosetting rate and percentage of multiplets

Rosetting rate was determined by microscopy and compared against the corresponding percentage of multiplets to determine correlation. A spread of FCR3S1.2R rosetting rates was generated by using varying concentrations of rosette-disrupting heparin, or by using patient sera with varying rosette-disrupting properties^11^.

**Figure 2:**
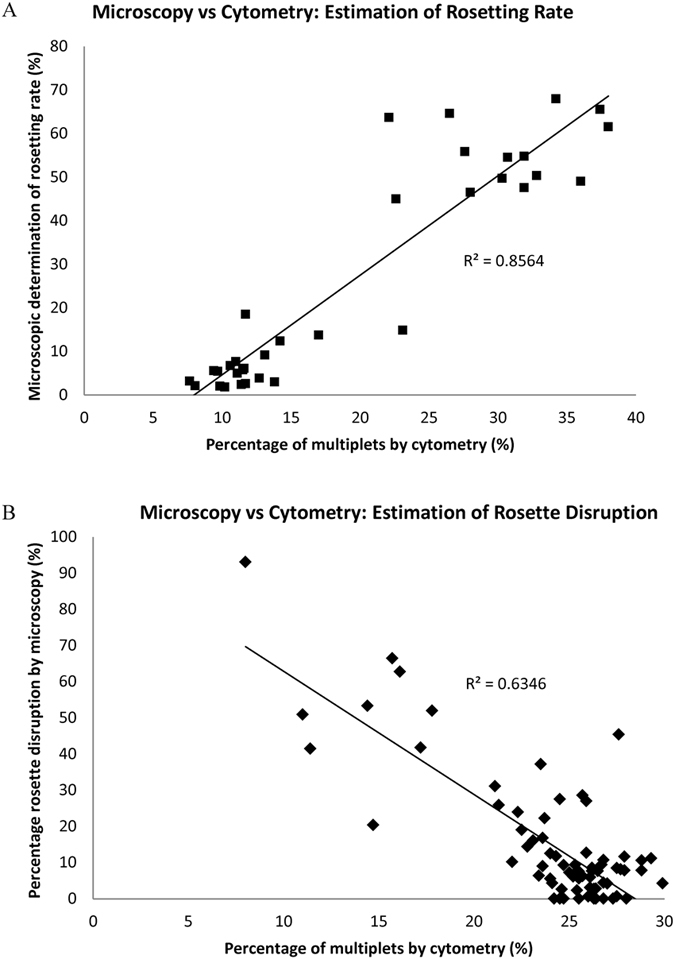
Correlation between rosetting rate and percentage of multiplets. (**A**) Rosetting FCR3S1.2R parasites were treated for 2 h with various concentrations of heparin (0–5 mg/mL). Samples were split and analysed by microscopy to determine rosetting rate (100–200 iRBC) or by flow cytometry to determine percentage of multiplets (N = 4). (**B**) Rosetting FCR3S1.2R parasites were treated for 1 h with sera from 74 Cameroon patients and percentage of multiplets was determined by cytometry. Published values of the corresponding rosette-disruption effect in respective patient sera, determined by microscopy, were used to determine correlation.

After 2 h incubation with 0–5 mg/mL of heparin, the rosetting rate and percentage of multiplets of FCR3S1.2R parasites were quickly determined (within the span of 1 h) by microscopy and cytometry respectively (Fig. 2A). Pearson’s correlation coefficient (R) of 0.925 (R^2^ = 0.8564) suggested a strong linear relationship between these two variables.

Similarly, Pearson’s correlation between percentage of multiplets and published values of the level of serum-induced rosette disruption^11^ (the inverse of rosetting rate, determined by microscopy) was −0.80 (R^2^ = 0.6346) (Fig. 2B and Supplementary Table S1), supporting that cytometry can be used to detect changes in rosetting rate.

### Effect of antimalarial drugs on rosetting rate and PfEMP1-expression

Parasite rosetting has been associated with malaria complications and patients are usually treated with intravenous quinine (QN) or artesunate (AS). Using chloroquine (CQ) and atovaquone (AQ) for comparison, we investigated the effects of these compounds on the rosetting rates of S1.2R, PAvarO and R29, at therapeutic and supra-therapeutic levels.

**Figure 3:**
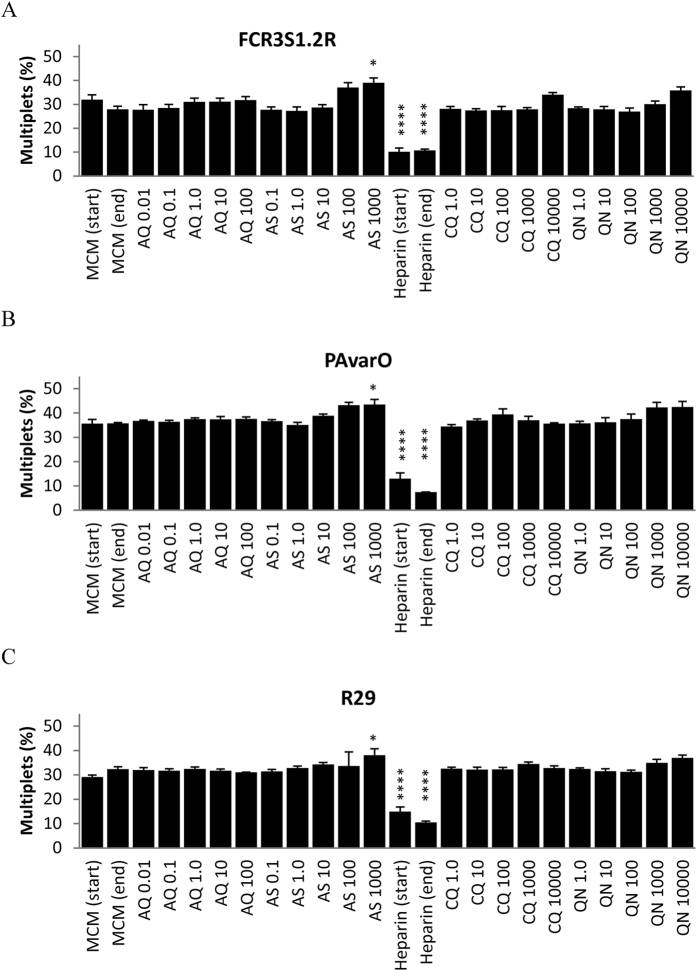
Effect of antimalarial drugs on rosetting. Rosetting trophozoites of (**A**) FCR3S1.2R, (**B**) PAvarO and (**C**) R29 were treated with various concentrations (between 0.01–10,000 μM) of atovaquone (AQ), artesunate (AS), chloroquine (CQ) or quinine (QN) for 12 h before being stained and analyzed by flow cytometry. Malaria culture media (MCM) and Heparin (10 mg/mL) controls from the start and at the end of the experiment were analyzed for comparison (*P < 0.05, ****P < 0.0001, N ≥ 3).

At the start of the experiment, an aliquot of the cultures, with or without 100 μg/ml heparin (MCM(start) and Heparin(start)), was kept at 4 °C and showed that the percentage of multiplets was maintained before and after the 12 h incubation period (MCM(end) and Heparin(end)) (Fig. 3A–C). Interestingly, none of the antimalarial drugs reduced rosetting in any of the parasite lines. Conversely, the slight increase in multiplets of all three parasites after treatment with 1000 nM of AS (P < 0.05) may suggest it actually increased rosetting. In comparison, the heparin controls showed a drastic reduction in percentage of multiplets from about 30% to 10% (P < 0.0001).

There was no detectable decrease in the expression of PfEMP1 after any of the treatments (Supplementary Fig. S1A–C), with the exception of a slight increase in anti-ITvar09 binding in R29 after treatment with 100 nM of AQ (P < 0.05). The persistence of PfEMP1 on iRBC surfaces and maintained rosetting levels suggest that the rate of spontaneous PfEMP1 degradation is low and that antimalarial chemotherapy is unlikely to affect parasite rosetting directly.

### Rosette disruption methods

**Figure 4:**
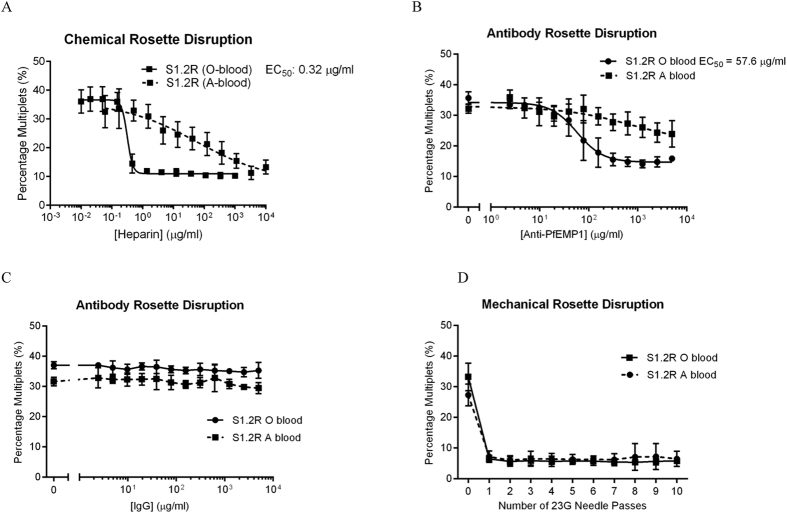
Effect of various rosette disruption methods on percentage of multiplets. Rosetting FCR3S1.2R parasites cultured in O+ or A+ erythrocytes were subject to various rosette disruption methods and analysed by flow cytometry. Cells were (**A**) treated with heparin (N = 7), (**B**) co-incubated with anti-PfEMP1 IgG (N = 3) or (**C**) non-immune IgG (N = 3) or (**D**) mechanically disrupted by passage through 23G needle (N = 5).

Parasites were treated with varying concentrations of heparin for 2 h prior to generate a dose-response curve. While non-rosetting S1.2NR and NF54CSA parasites showed a basal level of multiplets (around 10%, Supplementary Fig. S2), the percentage of multiplets was reduced in rosetting FCR3S1.2R, PAvarO and R29 parasites (from around 30% to 10%) at higher heparin concentrations (Fig. 4A and Supplementary Fig. S2). The effective concentration 50% values (EC_50_), a measure of the drug concentration required to halve the rosetting rate, were between 0.22–2.10 μg/ml.

As FCR3S1.2R parasites are known to form tighter and larger rosettes when cultured in blood group A^6^, we also assayed the effect of heparin on these rosettes. Although the EC_50_ value could not be determined (poor-fitting curve), a higher concentration of heparin is clearly required to disrupt rosettes (Fig. 4A). Similarly, blood group O rosettes were sensitive to anti-PfEMP1 antibody disruption while blood group A rosettes were resistant (Fig. 4B). Non-immune IgG did not reduce the percentage doublets in either cultures (Fig. 4C). In comparison, mechanical disruption (trituration with a 23G needle) was equally effective against both blood group O and A rosettes, with percentage multiplets reaching baseline values of about 10% (Fig. 4D).

### Rosette disruption by modified heparins and other sulfated glycosaminoglycans

To compare blood group-dependent sensitivity to other glycosaminoglycans, FCR3S1.2R parasites grown in group O or A erythrocytes were treated for 2 h with 100 μg/ml of unmodified or modified bovine heparin with varying levels of sulfation (Supplementary Table S2). Heparan sulfate, CSA, CSC, keratan sulfate and *E. coli* K5 were also included as controls.

**Figure 5:**
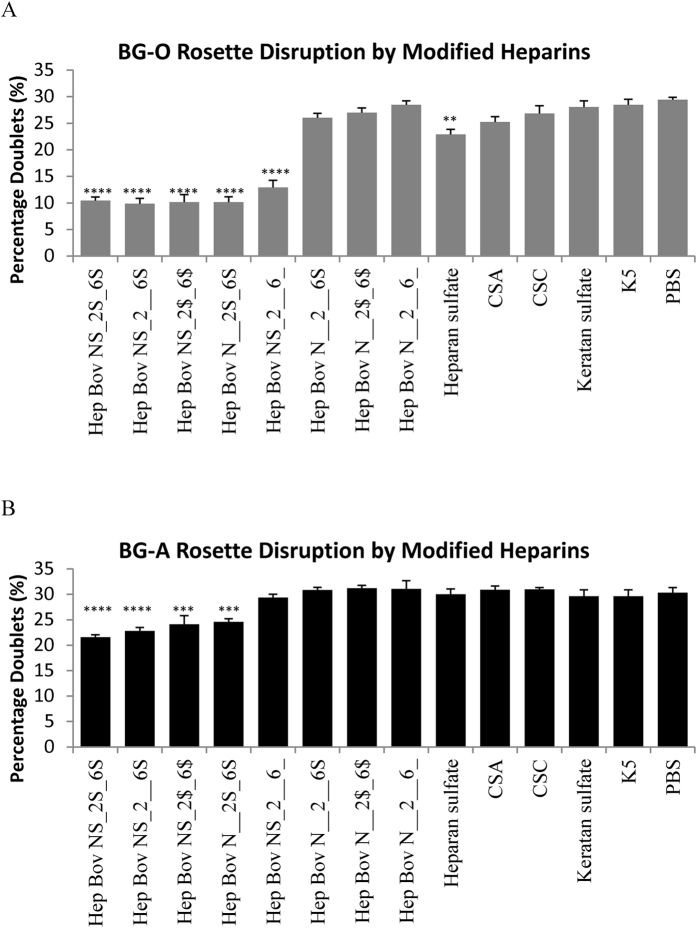
Rosette-disruption properties of modified heparins and other glycosaminoglycans. Rosetting FCR3S1.2R parasites grown in (**A**) O+ or in (**B**) A+ erythrocytes were treated for 2 h with 100 μg/ml of Bovine-sourced heparin (Hep Bov) that was unmodified (NS_2S_6S), 2-*O*-desulfated (NS_2__6S), partially 2-*O*, 6-*O* desulfated (NS_2$_6$), *N*-desulfated (N__2S_6S), 2-*O*, 6-*O* desulfated (NS_2__6_), *N*, 2-*O* desulfated (N__2__6S), *N* desulfated and partially 2-*O*, 6-*O* desulfated (N__2$_6$), *N*, 2-*O*, 6-*O* desulfated (N__2__6__), Heparan sulfate, Chondroitin sulfate A (CSA), Chondroitin sulfate C (CSC), Keratan sulfate, *E. coli* cell wall polysaccharide K5 (K5) or PBS control. Cells were analysed by flow cytometry and the percentage of multiplets after treatment are indicated (**P < 0.01, ***P < 0.001, ****P < 0.0001 when compared to DMSO control, N ≥ 3).

As described before^12^, the level of heparin sulfation corresponded with blood group O rosette disruption (Fig. 5A). Highly sulphated heparins (1.3–2.7 sulfate groups per disaccharide) showed a near-complete disruption with the proportion of multiplets decreasing from 30% to the baseline 10% (*P* < 0.0001). Conversely, highly de-sulfated heparins and low-sulfated polysaccharides (0–1.1 sulfate groups per disaccharide) had little or no activity.

Interestingly, blood group A rosettes were only partially sensitive to highly sulfated heparins (1.6–2.7 sulfate groups per disaccharide), with the reduction in percentage of multiplets decreasing only to 22–25% (Fig. 5B). Notably, 2-*O* 6-*O* desulfated heparin (1.1 sulfate groups per disaccharide) had no activity, suggesting higher requirement of glycosaminoglycan sulfation for blood group A rosette disruption.

### Drug library screening for blood group A rosette disruptors

Since blood group A is associated with disease severity and blood group A rosettes demonstrated heparin resistance, we specifically screened for compounds effective against rosettes of this blood group. To ascertain that the cytometry-based assay would be suitable for HTS, the Z-factor (Z’) was calculated to give an indication of the assay’s signal-noise ratio (ensuring significant separation of positive and negative controls to facilitate hit selection). With 10 mg/mL of heparin as a positive control, the Z’ was calculated to be 0.57 (validation over three experiments, data not shown) indicating suitability for HTS.

We therefore proceeded to perform a pilot screen on three compound libraries, and achieved a throughput of almost two 96-well plates per hour and an average Z’ (±standard deviation) of all 32 screening plates of 0.71 (±0.099).

Although no hits surfaced which were comparable to the heparin controls, 23 potential hits showed a slight reduction in percentage of multiplets compared to the vehicle controls: 17 compounds from the Prestwick screen that were below three standard deviations from the mean of negative controls of each plate, 5 compounds from the Asinex PPI screen that were below the threshold of 25% multiplets and 1 compound from the Malaria Box screen that was at the threshold of 25%. (Supplementary Figs S3–S5)

These 23 potential hits were cherry-picked and assayed again at 10 μM. In addition, five recently identified anti-sequestration compounds (from a biochemical screening of 10,000 ChemBridge DIVER-Set library compounds) were included^13^. The latter were assayed at 100 μM in accordance with the published study, with the following ChemBridge IDs were 5210644, 5210653, 5306995, 6917839 and 9121948. Five analogs of 5210653 (5210645, 5210646, 5210656, 5253203 and 7089200) and five analogs of 6917839 (6365236, 7951715, 9195948, 9277331 and 23605617) were also assayed.

**Figure 6:**
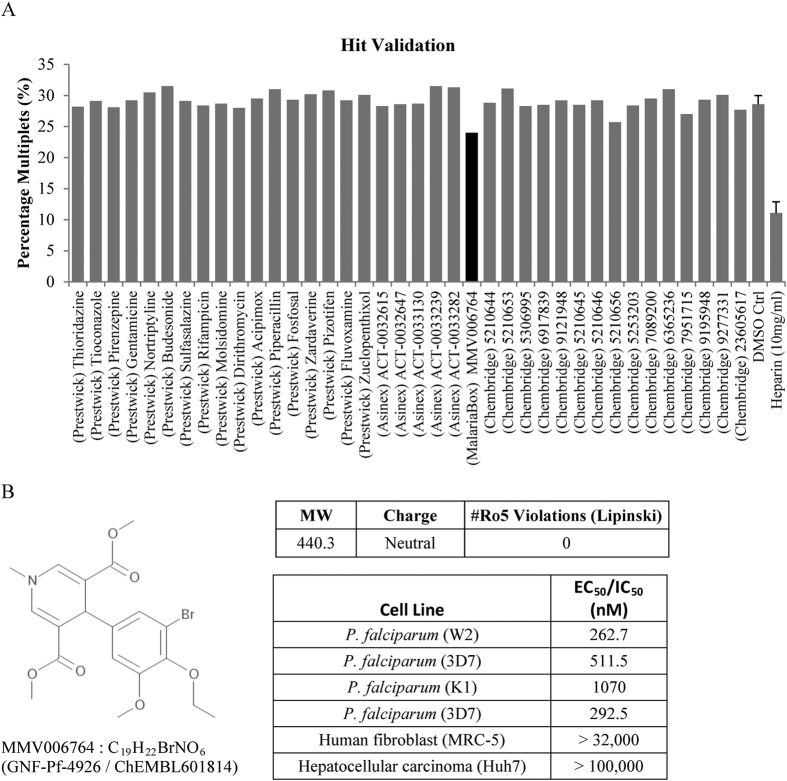
Hit validation and screening of ChemBridge compounds. (**A**) Hits from the Prestwick, Asinex Protein-Protein Interaction (PPI) and Malaria Box library screens were re-screened at 10 μM together with selected compounds from the ChemBridge drug library (100 μM), DMSO (0.1%) and Heparin (10 mg/mL) controls. Rosetting FCR3S1.2R parasites cultured in A+ erythrocytes were stained and treated for 2 h with test 10 μM compounds before flow cytometry analyses. (**B**) Compound properties of MMV006764 from ChEMBL database are tabled here including molecular weight (MW), charge, Lipinsky rule of 5 violations (#Ro5), effective concentration 50% (EC_50_) and inhibitory concentration 50% (IC_50_) values.

Of these 38 compounds, only MMV006764 showed a reduction in rosetting rate that was below two standard deviations from the mean of the DMSO control (Fig. 6A). Based on details available from the ChEMBL database, this compound from the Malaria Box is an uncharged molecule with molecular weight of 440.3 g/mol, no “Lipinsky rule of five” violations, established anti-plasmodial activity and low cytotoxicity against two human cell lines (Fig. 6B).

### Comparing MMV006764 and analogs

MMV006764 was purchased directly from a supplier, along with 25 structural analogs (62–98% similarity, Supplementary Table S3) and dose-response curves were generated. Of these, MMV006764 showed the most prominent effect (Supplementary Fig. S6). Though the extent of reduction was modest, MMV006764 concentrations between 30–250 μM resulted in a consistent reduction in the percentage of multiplets.

### Confirmation of MMV006764 rosette disruption activity

**Figure 7:**
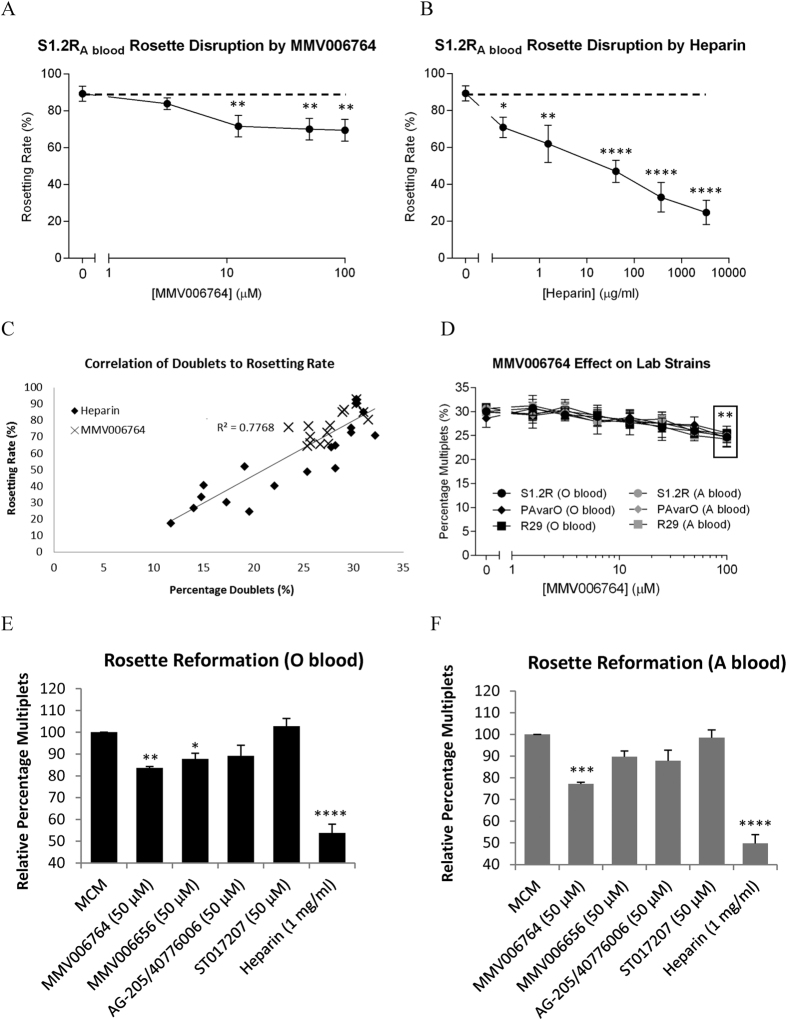
Validation of MMV006764 rosette disruption activity. Rosetting FCR3S1.2R parasites cultured in blood group A erythrocytes were treated for 2 h with various concentrations of (**A**) MMV006764 or (**B**) Heparin and the samples split for microscopic enumeration or (**C**) flow cytometry for correlation. (Between 150–300 late-stage parasites were counted in each microscopy sample, *P < 0.05, **P < 0.01, ****P < 0.0001, N = 3) (**D**) Cultures of rosetting FCR3S1.2R, PAvarO and R29 grown in blood group O+ or A+ erythrocytes were stained and treated with different concentrations of MMV006764 for 2 h before flow cytometry (**all points in box compared to untreated P < 0.01, N = 6, details of P-values in Supplementary Table S4). Rosettes of FCR3S1.2R grown in (**E**) blood group O+ or (**F**) A+ erythrocytes were dispersed by trituration and allowed to reform spontaneously in MCM, with or without 50 μM of MMV006764, MMV006656, AG-205/40776006 and ST017207, or 1 mg/ml of heparin.

Microscopy confirmed MMV006764 reduced rosetting rate from about 90% to 70% (Fig. 7A). In comparison, rosetting rate was reduced to about 20% with 3.3 mg/mL of heparin (Fig. 7B). Again, correlation between FCR3S1.2R rosetting rate and percentage of multiplets was strong (R^2^ = 0.7768, Fig. 7C) suggesting that the relationship between these two parameters was valid even in blood group A rosettes.

Whether cultured in blood group O or blood group A erythrocytes, rosetting FCR3S1.2R, PAvarO and R29 parasites all showed a similar reduction in the percentage of multiplets, from around 30% in the untreated controls to around 25% with 100 μM of MMV006764 (P < 0.01, Fig. 7D and Supplementary Table S4). This corresponded to about a 20% reduction in rosetting rate as determined by microscopy (P < 0.05, Supplementary Fig. S7).

The potential for a synergistic effect between MMV006764 and heparin was also investigated, but increasing levels of MMV006764 did not significantly reduce the concentration of heparin required to completely disrupt blood group A or O rosettes (Supplementary Fig. S8).

The effect of MMV006764 and three of its analogs (MMV006656, AG-205/40776006 and ST017207) on PfEMP1 var60 levels was also measured on FCR3S1.2R parasites but no effects were observed in either blood group O or group A erythrocytes (Supplementary Fig. S9).

Additionally, the effect of MMV006764 and its analogs were tested for their ability to prevent mechanically-dispersed rosettes from spontaneously reforming (Fig. 7E). In FCR3S1.2R cultures grown in blood group O erythrocytes, both MMV006764 and MMV006656 demonstrated a slight reduction in the percentage of multiplets compared to the MCM control (P < 0.01 and P < 0.05 respectively) whereas the heparin control showed a more pronounced reduction (P < 0.0001). In comparison, only MMV006764 significantly reduced the percentage of multiplets (P < 0.001) when the same parasites were cultured in blood group A erythrocytes. Both AG-205/40776006 and ST017207 did not show any effect on preventing rosette reformation. Together, the data suggests that MMV006764 is partly able to reduce rosette reformation similarly in both blood group O and group A, whereas MMV006656 appeared to have an effect only against blood group O rosette reformation.

## Discussion

Rosetting has been associated with malaria complications and is recognized to contribute to microvasculature occlusion^3^,^4^,^14^,^15^,^16^. Though the benefits of rapidly dispersing rosettes to restore microcirculation are obvious, no licensed drug exists to meet this therapeutic niche. The determination of rosetting rate typically involves a microscopist counting the proportion of rosetting trophozoite- or schizont-infected cells^17^. Not surprisingly, screening efforts to identify rosette-disrupting compounds for the treatment of malaria complications have never been undertaken. We made use of cytometry to detect changes in rosetting rate by using multiplet discrimination^18^,^19^,^20^. When aggregates of two or more cells (called “doublets” or “multiplets”) are simultaneously interrogated by the cytometer, there is a loss of proportionality between the signal area and signal height. These events can be quantified to give an indication of changes in rosetting rate.

Though there was a strong association between the percentage of multiplets and rosetting rate of *P. falciparum*, the numbers did not match perfectly. Even when rosettes were absent by microscopy (completely dispersed by heparin), the percentage of multiplets was approximately 7–10%, suggesting that this baseline value could correspond to the percentage of multiplets that occur spontaneously during cytometry. Conversely, when rosetting rate was almost 70% (in the absence of heparin), the percentage of multiplets was only 40%, suggesting either that some of the rosettes could not be detected or may have been dispersed during passage through the cytometer.

Though cytometry may not precisely determine rosetting rate, this is compensated by its throughput. A microscopist might perform 100 assays a day by using a synchronous culture at high parasitemia (counting 200–300 iRBC per assay) but the same number of samples can be screened within an hour (accounting 2500 iRBC per assay) even using a mixed-stage culture at low to moderate parasitemia. The throughput and reliability of cytometry allowed us to explore the effect of antimalarial chemotherapy on rosetting.

Despite the clear benefits of using artesunate over quinine in the management of complicated malaria^21^, few studies have investigated how artesunate or other antimalarial compounds directly affect the abundance of sequestration-mediating proteins on the surface of iRBCs or on rosetting rate. Our results suggest that the amount of PfEMP1 proteins expressed on the late-stage parasite’s host cell does not decrease even after 12 h treatment with supra-therapeutic levels of antimalarials. Rosetting rate was also unaffected suggesting that antimalarial chemotherapy itself may not be able to quickly resolve parasites sequestration in the host microvasculature. This agrees with another study showing that drug-inactivated parasites continue to cytoadhere 24 h after treatment^22^. Since drug-inactivated iRBC do not mature or rupture *in vivo*, it is conceivable that such cells continue to occlude microvasculature until depleted by the host’s immune cells. This delay in restoring microcirculation exacerbates tissue damage and highlights the need to develop drugs that directly and rapidly de-sequester iRBCs.

Heparin has long been recognized to possess anti-sequestration activity and was once considered for treatment of malaria complications^12^,^23^,^24^. Though its anti-coagulant activity rendered it medically unsuitable^25^, *in vitro* rosette-disrupting properties of heparin have been well documented^12^ and we were able to observe a sharp decrease in the percentage of multiplets in blood group O rosettes. However, when applied to blood group A rosettes, the potency of heparin was drastically reduced. Similarly, antibodies against PfEMP1were much less effective in disrupting blood group A rosettes compared to blood group O. These results could be explained by recent findings that blood group A rosetting is mediated by a different protein family, the RIFINs^6^. In FCR3S1.2R, the RIFIN involved was identified to be encoded by PfIT_bin05750, which bound with high affinity to the blood group A antigen. Disruption of PfEMP1-heparan sulfate interactions by heparin may not provide sufficient reduction of affinity between iRBC and uRBCs to break up blood group A rosettes. The specific targeting of blood group A rosettes is of particular clinical significance since children with blood group A are more susceptible to severe malaria compared to those with blood group O^26^,^27^,^28^,^29^,^30^.

Using high throughput cytometry, we performed a screen for blood group A rosette-disrupting structures. From a pilot screen of 2693 compounds, only MMV006764 gave a consistent decrease in percentage of multiplets. This rosette-disruption activity was confirmed by microscopy, with rosetting rate decreasing by 20%. Importantly, MMV006764 also exhibited activity against two other rosetting laboratory parasite lines grown in group A erythrocytes. This activity against rosettes from different parasite backgrounds fulfills an important criterion for clinical relevance. Both blood group A and O rosettes were similarly sensitive to MMV006764, suggesting that a receptor-ligand interaction common to rosetting in both blood groups may be the target and further work will be required to identify its mechanism of action.

MMV006764 represents the first small molecule inhibitor of parasite rosetting. Though modest in its activity, MMV006764 could nonetheless reduce microvasculature blockage sufficiently to improve tissue perfusion and impact patient prognosis.

Together with rosette-disrupting activity, this compound from the Malaria Box collection also has sub-micromolar antimalarial activity and a favorable cytotoxicity profile. Being a small uncharged molecule with no violations to Lipinsky’s “rule of five”, MMV006764 could potentially be developed for oral/rectal formulation. This would allow it to be administered in a resource-poor setting without the need for intravenous injection (the necessary route of administration for highly charged glycosaminoglycan-based therapies) and would be especially beneficial for patients without hospital access.

Patients with malaria complications often deteriorate rapidly and succumb before reaching the hospital. Even those who do manage have a poor prognosis since prolonged hypoxic tissue damage could be irreversible. As such, there is a need to develop easily-administered treatments for patients presenting early signs of sequestration-related complications *en route* to hospital. Candidate drugs should be able to reverse parasite sequestration (not only prevent it) and will undoubtedly need to be administered in combination with potent antimalarials. However, given that MMV006764 has both anti-sequestration and anti-parasitic effects, this dual advantage could be synergistic in reducing morbidity and mortality. Further analysis of ADME as well as toxicology studies will be required to ascertain its therapeutic potential.

More extensive screening efforts could uncover yet other candidate structures, and the cell-based phenotype assay presented here provides a suitable platform for doing so. With its ease of use (no specialized reagents, transfectant cell lines or recombinant proteins required), minimal assay cost (no proprietary technology involved), physiological relevance (cell-based assay performed in 10% serum), simplicity (no centrifugation or washing steps, short assay duration), reliability (Z’ > 0.5) and good throughput (200 compounds/hr), this assay allows small academic laboratories and large pharmaceuticals alike to screen for urgently-needed anti-sequestration compounds.

The spread of artemisinin resistant malaria means that there is little left in our arsenal of effective antimalarials. Despite prolonged parasite clearance times and higher possibility of complications, there remains no treatment to rapidly dislodge sequestered parasites and restore microcirculation. We report here the first assay amenable for the high-throughput identification of rosette-disrupting compounds. A pilot screen identifies Malaria Box compound MMV006764 as a potential candidate. Though modest in effect, MMV006764 was able to disperse heparin-resistant blood group A rosettes to a similar degree as heparin-sensitive blood group O rosettes. Coupled with its antiplasmodial activity, MMV006764 should be prioritized in the drug development pipeline especially given its potential to be easily administered in a resource-limited setting. MMV006764 represents the first small molecule inhibitor of rosetting and provides a starting point for the development of field-ready drugs that directly addresses the issue of malaria complications while simultaneously killing parasites.

## Methods

### Parasite culture

Rosetting FCR3S1.2R, PaloAlto_varO_ (PAvarO) and R29 *P. falciparum* parasites, as well as non-rosetting FCR3S1.2NR and NF54CSA parasites were cultured in O+ or A+ erythrocytes with 10% A+ serum^6^,^17^. Rosetting parasites were Ficoll-enriched approximately once a month and all cultures were sorbitol synchronized weekly^17^.

### Glycosaminoglycan preparations

Heparin (Porcine intestine, Sigma-Aldrich catalog number H3393) was dissolved in PBS to 100 mg/mL (approximately 20,000 heparin USP units/ml). Unmodified and modified heparins (bovine) were synthesized as described elsewhere^31^, along with heparan sulfate and *E. coli* cell wall polysaccharide K5^32^. Chondroitin sulfate A (CSA), chondroitin sulfate C (CSC) and keratan sulfate (from bovine trachea, cartilage and cornea respectively) were purchased from Sigma-Aldrich (catalog number for CSA is C9819 while CSC and keratin sulfate have been discontinued). Details are summarized in Supplementary Table S2.

### Staining reagents and drugs

Dihydroethidium (DHE, Invitrogen) was dissolved in DMSO to 1 mg/mL and Hoechst 33342 (Invitrogen) was dissolved in dH_2_O to 200 μg/ml. Anti-PfEMP1 polyclonal goat IgG production and purification is described elsewhere^33^. Non-immune goat IgG (Jackson ImmunoResearch) and AlexaFluo488-conjugated rabbit anti-goat IgG (Invitrogen) were purchased. Chloroquine (CQ), quinine (QN), artesunate (AS) and atovaquone (AQ) (Sigma-Aldrich catalogue numbers C6628, Q1878, A3731, A7986 respectively) were dissolved in PBS or DMSO to 10 mM.

### Parasite staining procedures

Co-staining with Hoechst and DHE is described previously^10^, but anti-CD45-APC was excluded and concentration of Hoechst and DHE was adjusted to 10 μg/ml and 5 μg/ml respectively. 5 μL of a 10× Hoechst-DHE staining solution were added to wells containing 45 μL of cultures and incubated for 1–2 h. Immunostaining of PfEMP1 was performed by incubating cells with 100 μg/ml of anti-PfEMP1 IgG for 1 h at room temperature (RT). Cells were washed in PBS and resuspended in Alexa488 anti-goat IgG (1:200) with Hoechst-DHE for another hour.

### Microscopy

Rosetting rate was determined with a Nikon Optiphot2 fluorescence microscope under 40× magnification^17^ while cytometry-sorted rosettes were viewed with a Nikon Eclipse 80i fluorescence microscope under 100× magnification.

### Flow cytometry

The BD FACSVerse flow cytometer equipped with plate reader was used, with FlowJo (v10) for analyses. Gating was performed in the following succession: FSC-SSC to exclude debris, double-positive for Hoechst and DHE to select late-stage parasites and FSC-Area– FSC-Height to count multiplets. Data from 2500 late-stage parasites were collected for all assays. The BD FACSVantage Cell Sorter was used in the sorting of multiplets for microscopy.

### Rosette disruption by Cameroon patient sera

Cameroon patient sera collection procedure and rosette disruption activity has been described before^11^. FCR3S1.2R cultures were resuspended in 5× diluted patient sera, incubated for 1 h at RT and stained with Hoechst-DHE prior to cytometry.

### Rosette disruption methods

For chemical- and antibody-mediated rosette disruption, 50 μL of cultures resuspended in equivolume of the test solution, 10× DHE-Hoechst added, and incubated at RT for 2 h. To investigate the effects of mechanical rosette disruption, Hoechst-DHE pre-stained cultures were diluted to 0.2% hematocrit and passed through a 23G blunt-end needle using a 5 mL syringe. After each pass, 200 μL of culture was aliquoted into separate wells. Immediately after the tenth passage these wells were analyzed by cytometry to ensure that rosettes did not reform spontaneously.

### Rosette reformation assay

FCR3S1.2R parasites were stained with Hoechst-DHE, resuspended in MCM containing various test compounds to 0.2% hematocrit, triturated three times and left to incubate for 1 h at 37 °C for rosettes to reform. Thereafter, 150 μL was transferred to a U-bottom 96-well plate for cytometry.

### Library screening

Libraries screened include Prestwick Chemical Library of 1280 small molecules, a sub-set of 1008 compounds from the Asinex Protein-Protein Interaction (PPI) library, and the Malaria Box library (Medicines for Malaria Venture) of 390 compounds^34^. Library compounds (10 mM in DMSO) were stored at ambient temperature under nitrogen gas. Plating into a 96-well format was done by ECHO555 dispensing (Labcyte, USA) to a final screening concentration of 10 μM. A minimum of four solvent (DMSO) negative controls and four heparin (10 mg/mL) positive controls were added to each plate. At the start of the experiment, 50 μL of Hoechst-DHE pre-stained FCR3S1.2R cultures (grown in A+ erythrocytes) was added to each well, resuspended by vortexing briefly and left to incubate for 2 h at RT. Plates were then stored at 4 °C in a humidified container for up to 36 h prior to cytometry.

### Verification of screening hits, short-listed ChemBridge compounds and MMV006764 analogs

Potential hits were re-screened at 10 μM a second time. ChemBridge compounds from the DIVER-Set library were purchased directly from the vendor, dissolved in DMSO and screened at 100 μM. MMV006764 and its 25 analogs were purchased from SPECS, Vitas-M Laboratory or Maybridge (Supplementary Table S3), dissolved in DMSO and assayed in two-fold serial dilutions. These 25 analogs were a subset of 54 analogs identified from the ~125,000 compounds available at Karolinska High Throughput Center using tools within the Collaborative Drug Discovery database system based on ChemAxon fingerprints.

### Statistics

At least three independent experiments were performed except for drug screens and hit verification. Graphs show mean and standard error of mean. Statistics were generated with GraphPad Prism 6.04 (EC_50,_ ANOVA post-hoc analyses and paired t-test) or Excel (Z’ and Pearson’s R). EC_50_ values were obtained from four-parameter dose-dependent curve in GraphPad Prism.

### Ethics statement

All experiments were performed in accordance with relevant guidelines and regulations. Erythrocytes and serum used for parasite culture were collected from the Karolinska Hospital blood bank (ethical permit number: 2009/668-31/3) as approved by the Regional Ethical Review Board in Stockholm, Sweden. Cameroon patient sampling was approved by the Ministry of Basic Education, Republic of Cameroon (ethical permit number: G379/900) and the Regional Ethical Review Board in Stockholm, Sweden (ethical permit number: 2006/1323-13/1), with informed consent obtained from all subjects or guardians.

## Additional Information

**How to cite this article**: Ch’ng, J.-H. *et al*. Rosette-Disrupting Effect of an Anti-Plasmodial Compound for the Potential Treatment of *Plasmodium falciparum* Malaria Complications. *Sci. Rep.* **6**, 29317; doi: 10.1038/srep29317 (2016).

## Supplementary Material

Supplementary Information
