# Supplementary material for: Rosette-Disrupting Effect of an Anti-Plasmodial Compound for the Potential Treatment of Plasmodium falciparum Malaria Complications
Source: Sci Rep. 2016 Jul 11;6:29317. doi: 10.1038/srep29317 (PMC4941523; doi:10.1038/srep29317)
Supplement: Supplementary Information [file srep29317-s1.pdf]

Supplementary Figures for:

Rosette-Disrupting Effect of an Anti-Plasmodial Compound for the Potential Treatment of *Plasmodium falciparum* Malaria Complications

Jun-Hong Ch'ng, Kirsten Moll, Maria del Pilar Quintana, Sherwin Chun Leung Chan, Ellen Masters, Ernest Moles, Jianping Liu, Anders B Eriksson & Mats Wahlgren

Supplementary Figure S1

A

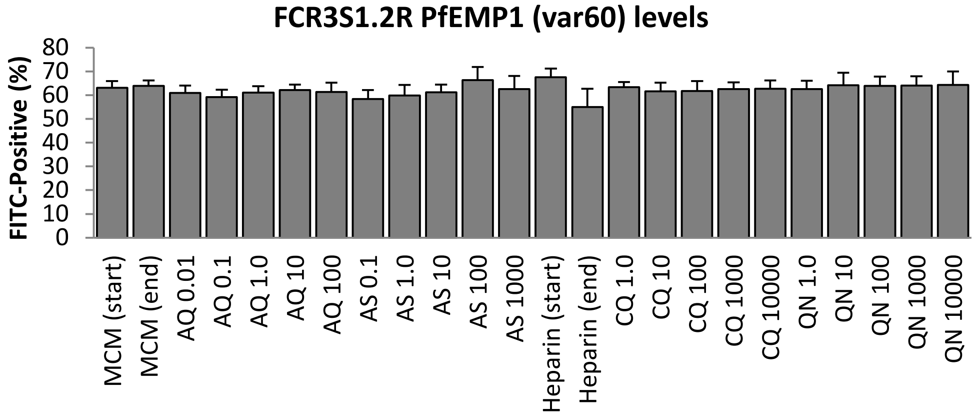

B

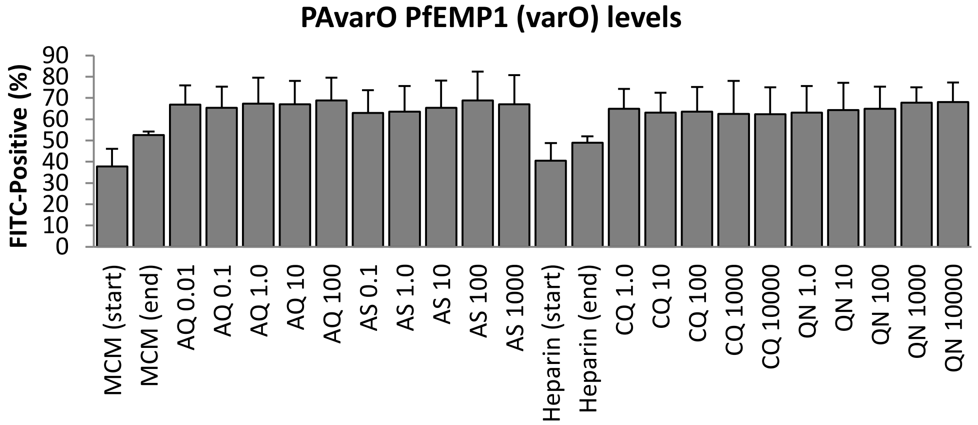

C

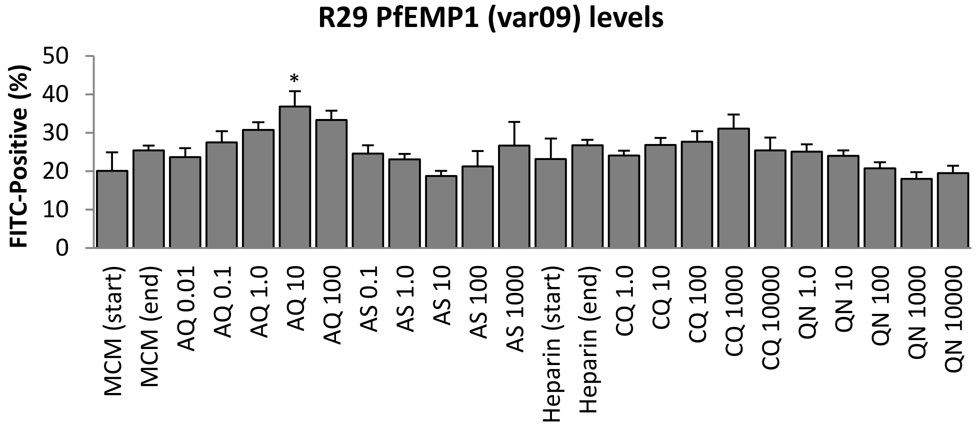

Supplementary Figure S1. Effect of antimalarial drugs on PfEMP1 levels. Rosetting trophozoites of (A) FCR3S1.2R, (B) PAvarO and (C) R29 were treated with various concentrations (between 0.01 – 10,000 µM) of atovaquone (AQ), artesunate (AS), chloroquine (CQ) or quinine (QN) for 12 h before being stained with respective polyclonal goat anti-PfEMP1 IgG and analyzed by flow cytometry. Malaria culture media (MCM) and Heparin (10 mg/mL) controls from the start and at the end of the experiment were analyzed for comparison (* $P < 0.05$, $N \geq 3$).

Supplementary Figure S2

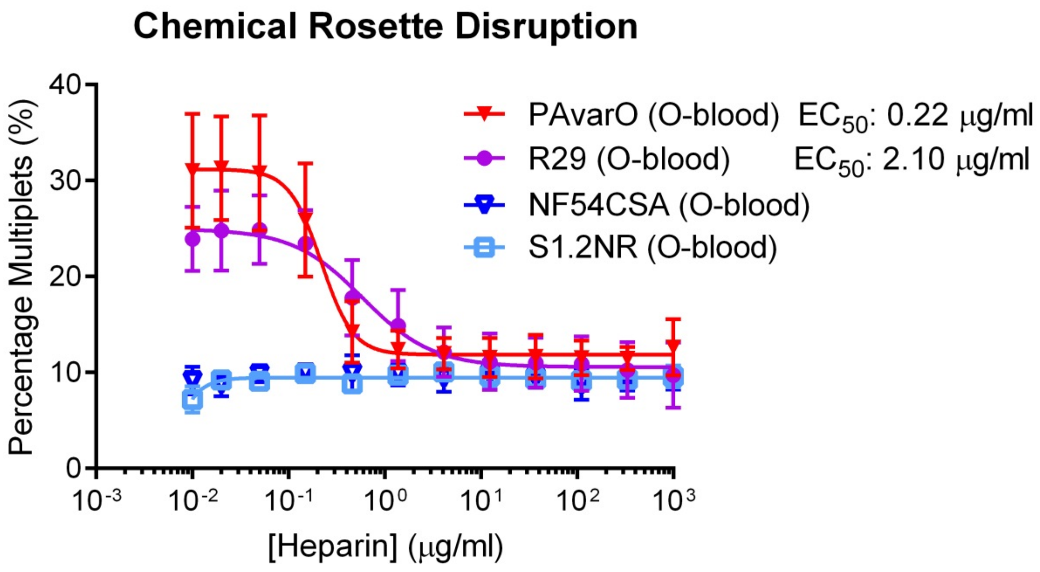

Supplementary Figure S2. Effect of heparin on percentage of multiplets. Rosetting PAvarO and R29 parasites, as well as non-rosetting NF54CSA and FCR3S1.2NR parasites, all cultured in O+ erythrocytes, were subject to various concentrations of heparin and the percentage of multiplets determined by cytometry (N ≥ 4).

Supplementary Figure S3

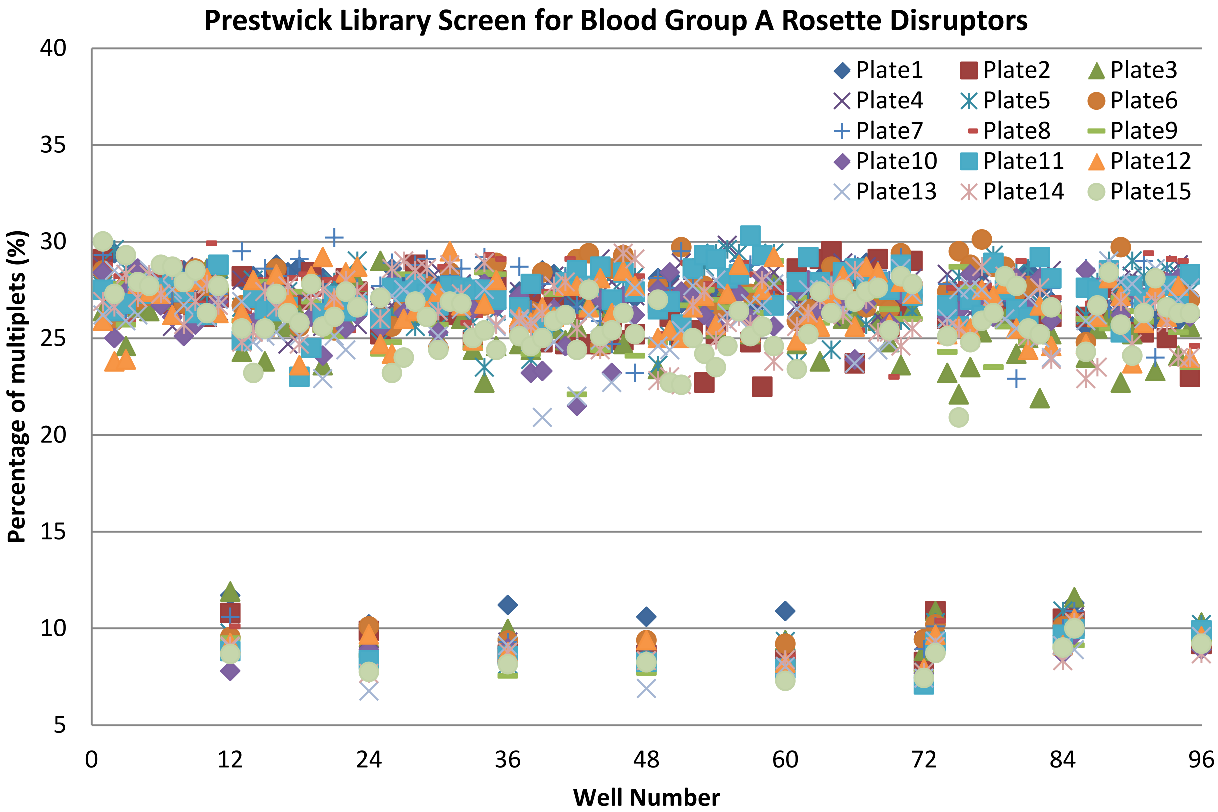

Supplementary Figure S3. Screening of Prestwick library of known drugs for rosette disrupting compounds. Rosetting FCR3S1.2R parasites cultured in A+ erythrocytes were stained and treated for 2 h with 10 µM of test compounds before flow cytometry. DMSO negative controls in column 1 (wells 1, 13, 25, 37, 49 and 61) and Heparin (10 mg/mL) positive controls in column 12 (wells 12, 24, 36, 48 and 60) of each plate. Non-rosetting S1.2NR parasites with DMSO control in wells 73 and 85 and with Heparin (10 mg/mL) in wells 84 and 96 of each plate.

Supplementary Figure S4

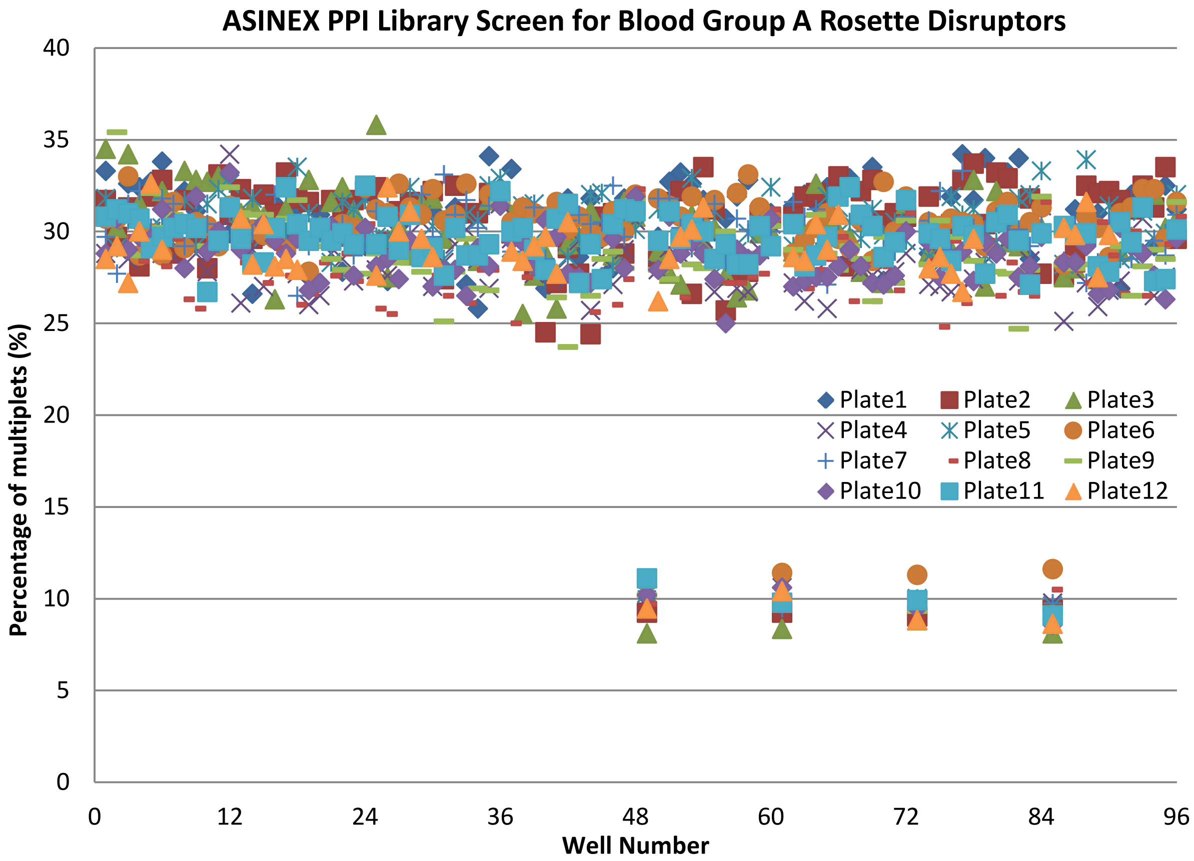

Supplementary Figure S4. Screening of Asinex PPI library for rosette disrupting compounds. Rosetting FCR3S1.2R parasites cultured in A+ erythrocytes were stained and treated for 2 h with 10 µM of test compounds before flow cytometry. DMSO negative controls in wells 1, 13, 25 and 37 while Heparin (10 mg/mL) positive controls were in wells 49, 61, 73 and 85 of each plate.

Supplementary Figure S5

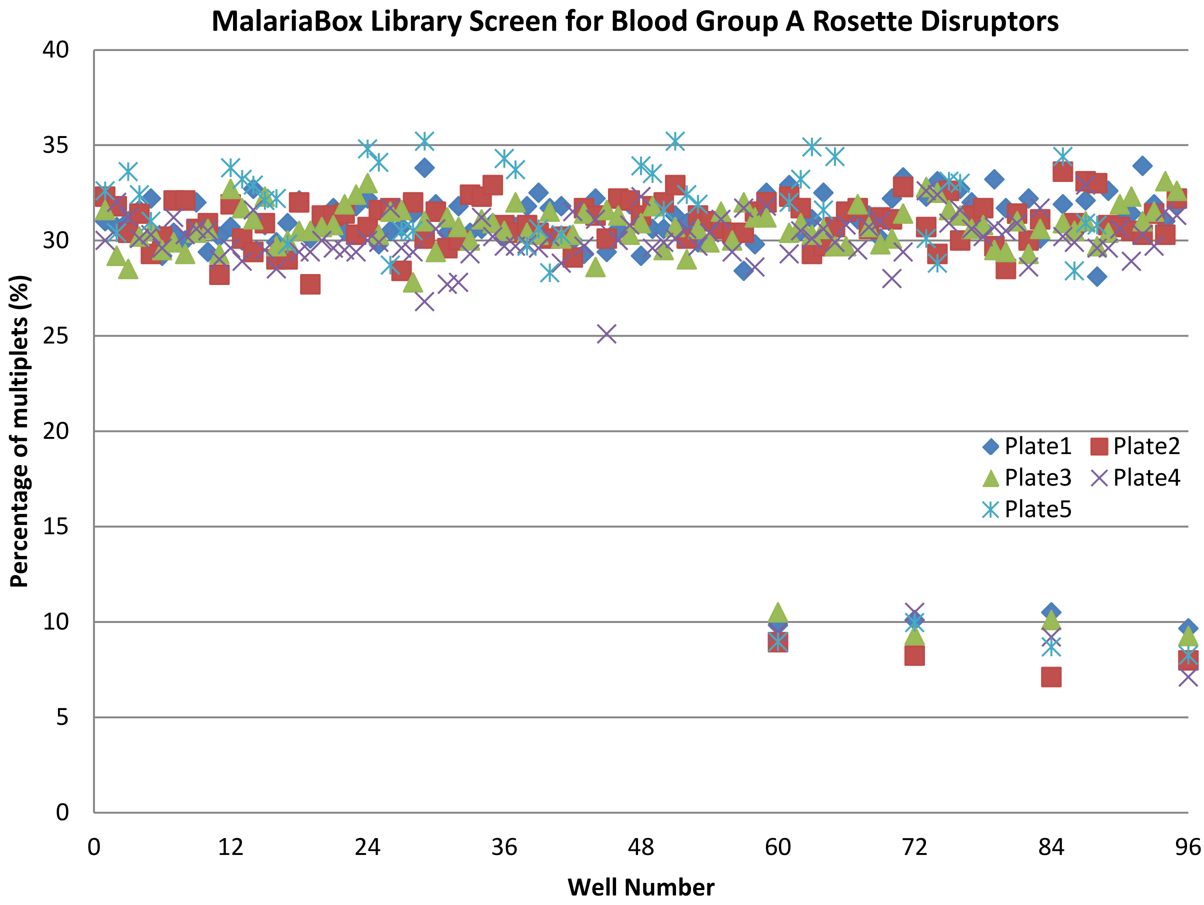

Supplementary Figure S5. Screening of Malaria Box library for rosette disrupting compounds. Rosetting FCR3S1.2R parasites cultured in A+ erythrocytes were stained and treated for 2 h with 10 µM of test compounds before flow cytometry. DMSO negative controls in wells 12, 24, 36, 48 and Heparin (10 mg/mL) positive controls in wells 60, 72, 84 and 96 of each plate.

Supplementary Figure S6

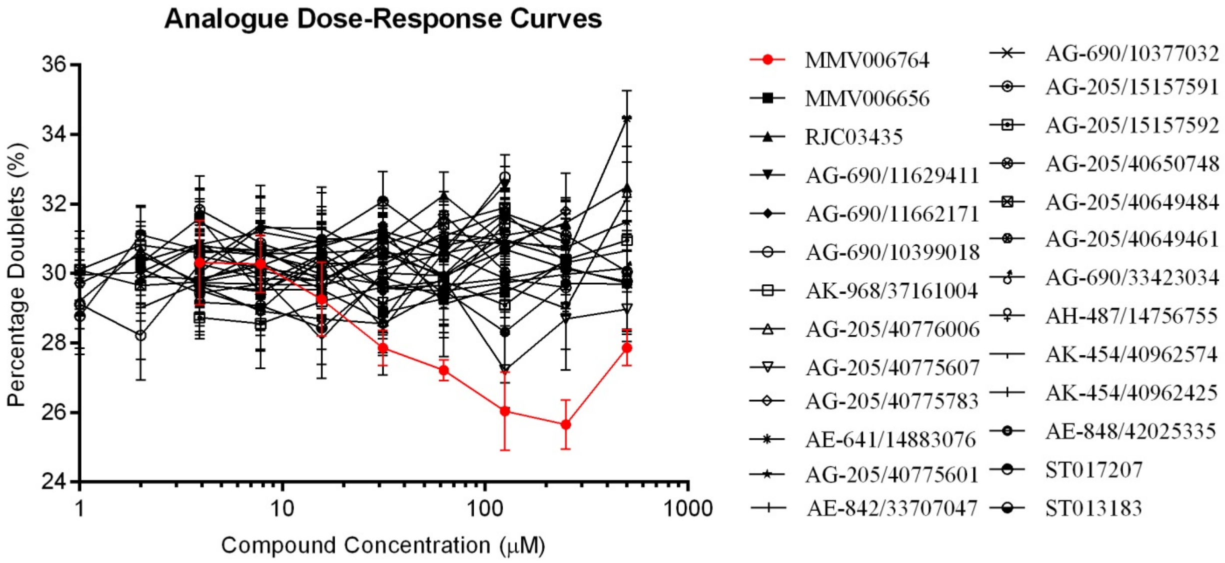

Supplementary Figure S6. Dose-dependent rosette disruption by MMV006764 and analogs. Rosette disruption activity of MMV006764 and 25 analogs (62-98% similarity) was determined using FCR3S1.2R parasites cultured in A+ erythrocytes. 8 two-fold serial dilutions of the various compounds were added to cultures, stained and incubated for 2 h prior to cytometry and the proportion of late-stage parasites presenting as multiplets was measured. MMV006764 demonstrated the highest rosette-disrupting effects and is highlighted in red (N = 5).

Supplementary Figure S7

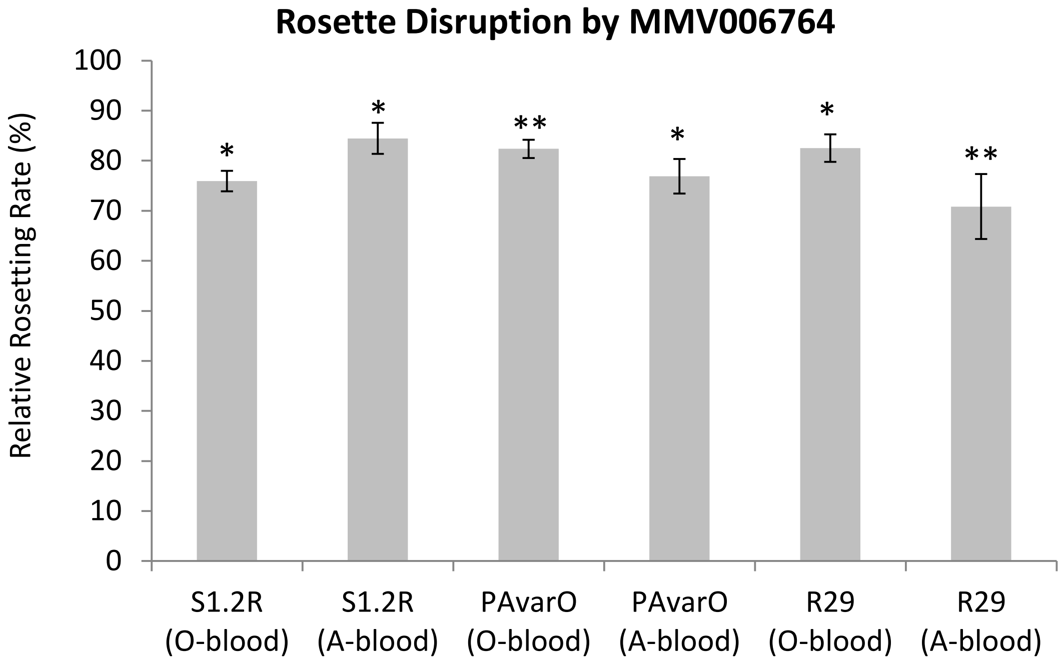

Supplementary Figure S7. Effect of MMV006764 on rosetting rate. Cultures of FCR3S1.2R, PAvarO and R29,grown in type O+ or A+ erythrocytes were treated for 2 hr with vehicle control (0.2% DMSO) or 100 µM of MMV006764. Samples were then stained with acridine orange and the rosetting rate quantified by microscopy by counting at least 300 late-stage parasites. Bars show the rosetting rate of each culture relative to the corresponding vehicle controls, with significant difference to the vehicle control determined by paired t-test (* $P < 0.05$, ** $P < 0.01$, $N \geq 3$).

Supplementary Figure S8

A

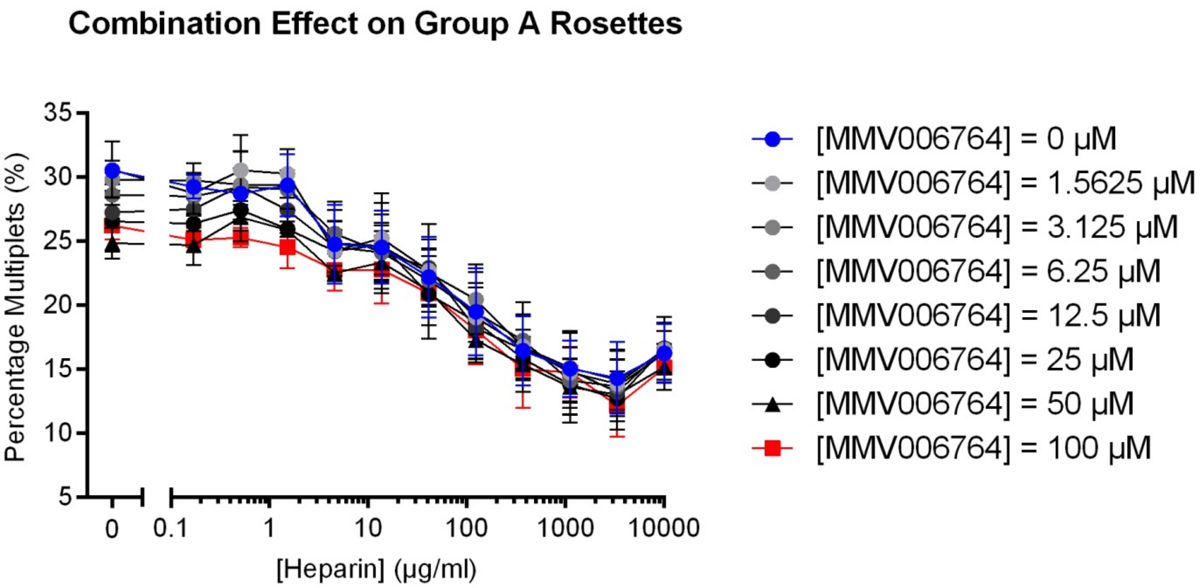

B

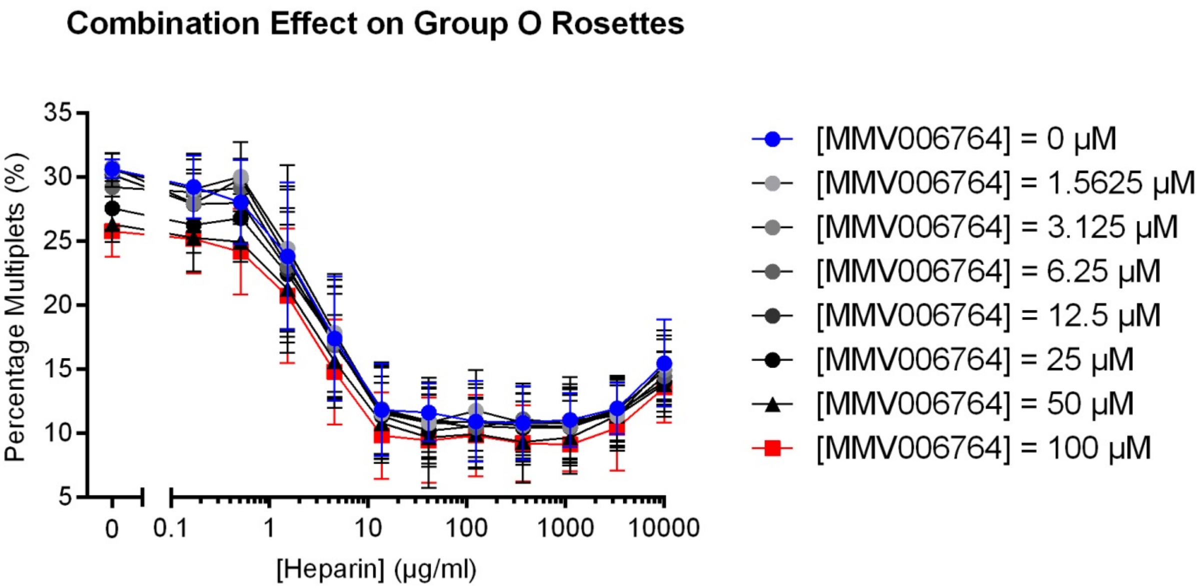

Supplementary Figure S8. Combinatory effect of Heparin and MMV006764. Percentage of multiplets of FCR3S1.2R parasites grown in (A) A+ erythrocytes (N=3) or (B) O+ erythrocytes (N=5) were determined after treatment with different combinations of heparin and MMV006764 concentrations.

Supplementary Figure S9
A

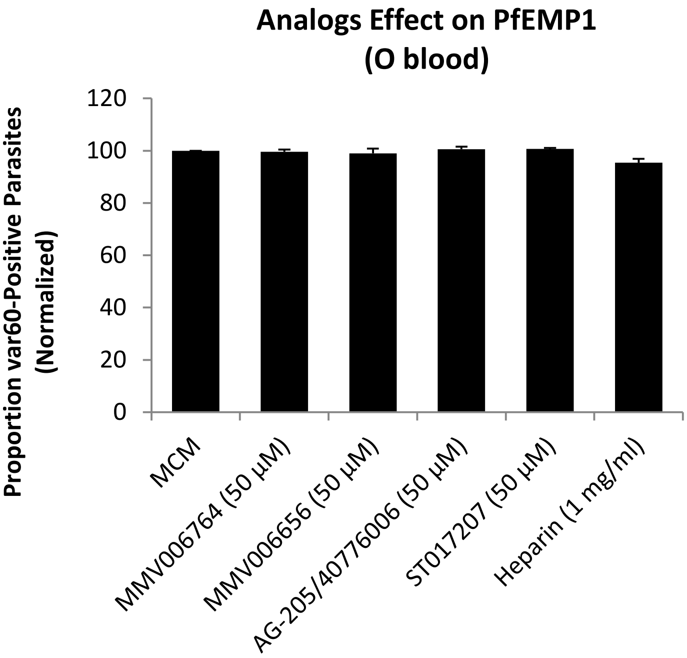

B

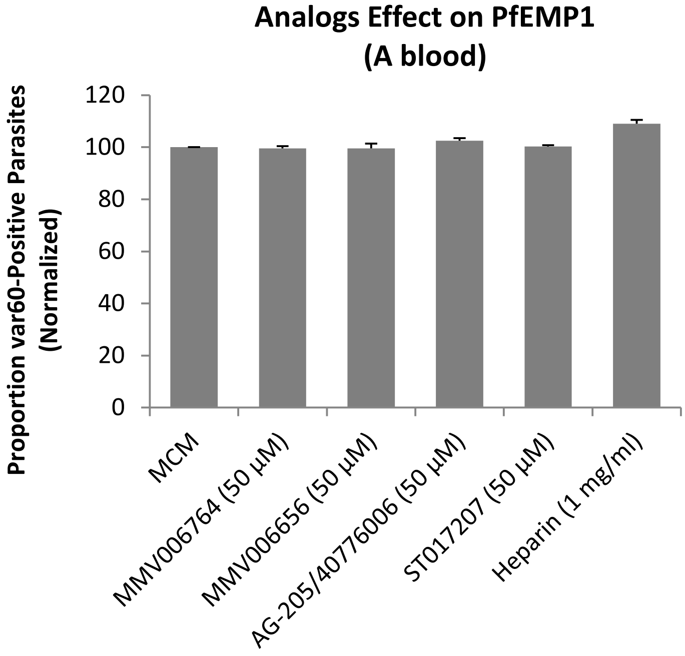

Supplementary Figure S9. Effect of MMV006764 analogs on PfEMP1 levels. FCR3S1.2R parasites grown either in (A) O+ erythrocytes (N=4) or (B) A+ erythrocytes (N=4) were treated for 2 h with different compounds before being washed and labelled with anti-var60 antibodies. The percentage of var60-positive iRBCs were normalized to the respective media-only controls (MCM).

Supplementary Tables for:

Rosette-Disrupting Effect of an Anti-Plasmodial Compound for the Potential Treatment of *Plasmodium falciparum* Malaria Complications

Jun-Hong Ch'ng, Kirsten Moll, Maria del Pilar Quintana, Sherwin Chun Leung Chan, Ellen Masters, Ernest Moles, Jianping Liu, Anders B Eriksson & Mats Wahlgren

Supplementary Table S1

Sample ID	% multiplets (Cytometry)	Disruption (Microscopy)
CBM001	28.0	0.0
CBM002	24.2	0.0
CBM003	24.7	0.0
CBM004	25.9	12.7
CBSr005	25.5	0.0
CBM006	26.4	2.7
CBM007	23.5	37.2
CBSa008	27.3	0.0
CBM009	22.0	10.2
CBM010	17.2	41.8
CBM011	11.4	41.5
CBM012	22.3	23.9
CBM013	11.0	50.9
CBM014	24.0	5.5
CBM015	26.2	1.1
CBM020	26.8	10.7
CBM021	25.3	9.3
CBM022	25.5	5.6
CBM023	25.4	2.3
CBM024	24.5	0.0
CBM028	27.0	4.2
CBM029	25.7	6.2
CBM031	23.6	9.0
CBM032	24.5	27.5
CBM033	26.5	7.7
CBSa035	27.9	7.8
CBM036	8.0	93.1
CBM037	26.2	7.6
CBM040	26.3	2.7
CBM041	17.8	52.0
CBM042	27.6	45.4
CBM043	14.7	20.4
CBM045	29.3	11.2
CBM046	24.7	9.3
CBSa047	28.8	7.8
CBM048	24.3	11.7
CBSa053	26.4	0.0

Sample ID	% multiplets (Cytometry)	Disruption (Microscopy)
CBM054	23.6	16.8
CBM056	26.3	0.0
CBSr060	25.4	7.6
CBM063	26.8	0.0
CBM064	25.5	7.5
CBSa065	21.1	31.1
CBM066	16.1	62.8
CBM068	15.7	66.4
CBM069	26.3	0.0
CBM072	14.4	53.3
CBM073	22.8	14.3
CBM074	23.7	22.3
CBM075	28.8	10.6
CBM077	27.5	0.6
CBM078	27.7	8.1
CBM079	25.7	28.6
CBM080	26.8	4.4
CBM081	25.9	27.0
CBM083	25.2	6.1
CBM084	24.2	0.0
CBM086	26.0	0.6
CBSa088	27.9	11.6
CBSa089	29.9	4.2
CBSa091	27.5	8.5
CBM138	24.1	4.3
CBM139	26.1	3.1
CBSc155	21.3	25.8
CBSa162	26.1	5.9
CBsa168	22.5	19.0
CBCs169	24.0	12.5
CBM170	23.4	6.4
CBSa171	26.7	9.4
CBM172	23.1	16.1
CBSa173	26.2	8.5
CBM174	24.5	0.0
CBSa175	24.6	2.6
CBSr176	25.0	7.2

Supplementary Table S1. Details of sera-induced rosette disruption. Break-down of sample identity and corresponding percentage of multiplets (determined by cytometry) and corresponding published values of percentage rosette disruption (determined by microscopy).

Supplementary Table S2

**Compound Name**	**Abbreviation**	**Source**	**N-sulfation (%)**	**2-O-sulfation (%)**	**6-O-sulfation (%)**	**# sulfate per disaccharide**
Unmodified heparin	(NS_2S_6S)	Bovine Lung	>95	80	88	2.7
2-*O*-desulfated heparin	(NS_2__6S)	Bovine Lung	>95	1	88	1.9
Partially 2-*O*, 6-*O* desulfated heparin	(NS_2$_6$)	Bovine Lung	95	61	10	1.7
N-desulfated heparin	(N__2S_6S)	Bovine Lung	0	76	85	1.6
2-*O*, 6-*O* desulfated heparin	(NS_2__6_)	Bovine Lung	>95	31	3	1.3
N, 2-*O* desulfated heparin	(N__2__6S)	Bovine Lung	0	17	88	1.1
N desulfated, partially 2-*O*, 6-*O* desulfated heparin	(N__2$_6$)	Bovine Lung	0	61	10	0.7
N, 2-*O*, 6-*O* desulfated heparin	(N__2__6__)	Bovine Lung	0	31	3	0.3
Heparan sulfate	HS	Porcine Intestine	variable	variable	variable	variable
Chondroitin sulfate A	CSA	Bovine Trachea	0	4-*O* sulfation variable	variable	variable
Chondroitin sulfate C	CSC	Bovine Cartilage	0	4-*O* sulfation variable	variable	variable
Keratan sulfate	KS	Bovine Cornea	0	0	variable	variable
K5 polysaccharide	K5	*E. coli* K5	0	0	0	0

Supplementary Table S2. Modified heparins and other glycosaminoglycans. Details of these compounds and their corresponding level of sulfation are described briefly.

Supplementary Table S3 (part 1 of 4)

Compound	Structure	Similarity (%)	Parent Library	InChIKey	Supplier
MMV006764	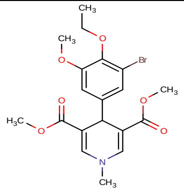	100.00	Malaria box	GHWXGIMICMXCPZ-UHFFFAOYSA-N	Vitas-M Lab
MMV006656	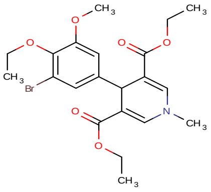	98.00	Malaria box	FBKIPJLWVIZHGV-UHFFFAOYSA-N	Vitas-M Lab
RJC03435	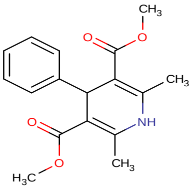	66.00	Maybridge	QDHIAGVDKRWZNF-UHFFFAOYSA-N	Maybridge
AG-690/11629411	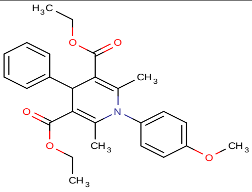	65.00	Specs	INNKVSIPMKSCPE-UHFFFAOYSA-N	Specs
AG-690/11662171	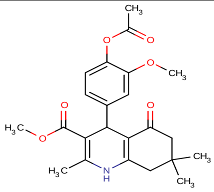	68.00	Specs	WJNSEFOSGMTATA-UHFFFAOYSA-N	Specs
AG-690/10399018	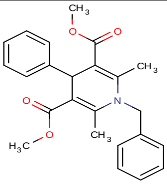	65.00	Specs	OLCZBDCFUSEEGV-UHFFFAOYSA-N	Specs
AK-968/37161004	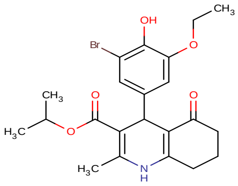	72.00	Specs	IDPMNNRUZGYOJG-UHFFFAOYSA-N	Specs
AG-205/40776006	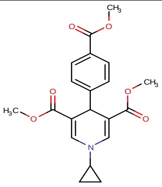	71.00	Specs	JLYXNYAVQUKLPI-UHFFFAOYSA-N	Vitas-M Lab
AG-205/40775607	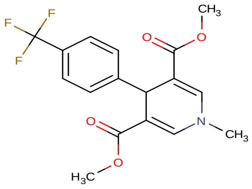	69.00	Specs	IVTYUXFCNQFJDN-UHFFFAOYSA-N	Specs

Supplementary Table S3 (part 2 of 3)

Compound	Structure	Similarity (%)	Parent Library	InChIKey	Supplier
AG-205/40775783	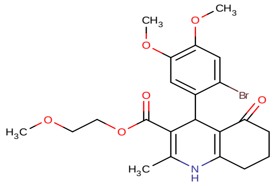	68.00	Specs	OZJPIUJNJPRFRU-UHFFFAOYSA-N	Vitas-M Lab
AE-641/14883076	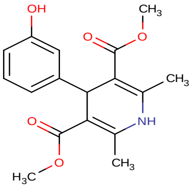	72.00	Specs	HWTUTDXHWQLDFZ-UHFFFAOYSA-N	Specs
AG-205/40775601	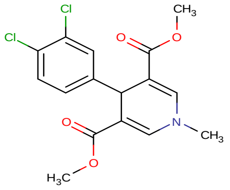	68.00	Specs	XKRSXVSBEJJYHO-UHFFFAOYSA-N	Vitas-M Lab
AE-842/33707047	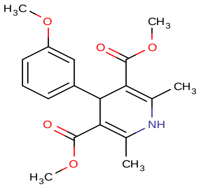	75.00	Specs	UVZJDLNWYRQSSL-UHFFFAOYSA-N	Specs
AG-690/10377032	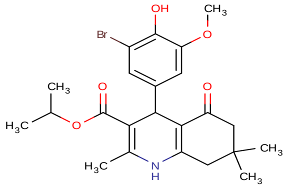	72.00	Specs	PAJOMZBUVDHRHG-UHFFFAOYSA-N	Specs
AG-205/15157591	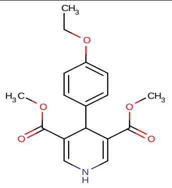	78.00	Specs	LSHZZZXMBXJLKJ-UHFFFAOYSA-N	Specs
AG-205/15157592	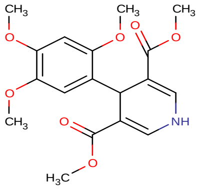	76.00	Specs	LDNBCCSMIHWEHV-UHFFFAOYSA-N	Specs
AG-205/40650748	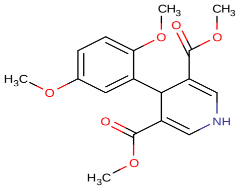	73.00	Specs	IOYLQTANSWXSNP-UHFFFAOYSA-N	Specs
AG-205/40649484	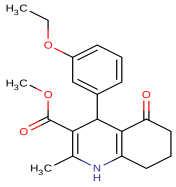	67.00	Specs	NNTZJFMOSKCNDU-UHFFFAOYSA-N	Vitas-M Lab

Supplementary Table S3 (part 3 of 3)

Compound	Structure	Similarity (%)	Parent Library	InChIKey	Supplier
AG-205/40649461	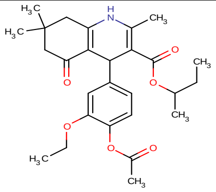	65.00	Specs	HWWIQHBGJZJJPS-UHFFFAOYSA-N	Vitas-M Lab
AG-690/33423034	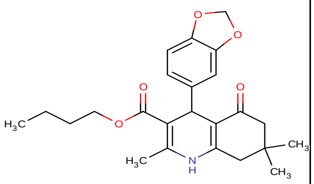	65.00	Specs	XBKXINSIFPYBIB-UHFFFAOYSA-N	Vitas-M Lab
AH-487/14756755	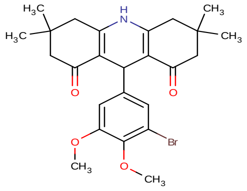	68.00	Specs	JOYLWBIQCXWMIO-UHFFFAOYSA-N	Specs
AK-454/40962574	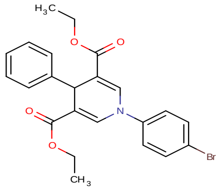	68.00	Specs	XRDIFEJDFBVJCB-UHFFFAOYSA-N	Specs
AK-454/40962425	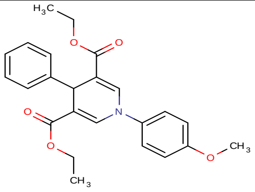	70.00	Specs	VIRJKNQQIURAIJ-UHFFFAOYSA-N	Specs
AE-848/42025335	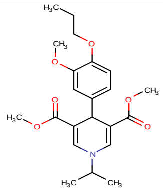	87.00	Specs	AQBVCVMJHJMUAA-UHFFFAOYSA-N	Specs
ST017207	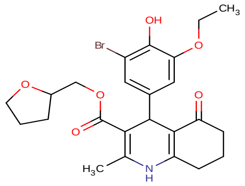	70.00	TimTec	MVJJOQINSDLWDI-UHFFFAOYSA-N	Vitas-M Lab
ST013183	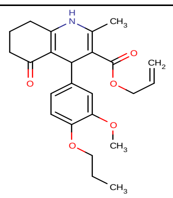	67.00	TimTec	YZCVORKVAHYZAI-UHFFFAOYSA-N	Vitas-M Lab

Supplementary Table S4

[MMV006764] (µM)	S1.2R (O-blood)	S1.2R (A-blood)	PAvarO (O-blood)	PAvarO (A-blood)	R29 (O-blood)	R29 (A-blood)
0						
1.5625	ns	ns	ns	ns	ns	ns
3.125	ns	ns	ns	ns	ns	ns
6.25	ns	*	ns	ns	ns	ns
12.5	ns	*	ns	ns	**	ns
25	**	**	ns	ns	**	*
50	***	****	ns	*	****	***
100	****	****	**	****	****	****

Supplementary Table S3. Detailed P-values for Figure 7D. Analyses by one-way ANOVA with Kruskal-Wallis Post-hoc was performed. Comparison for each concentration are made against the respective "no drug" control (* $P < 0.05$, ** $P < 0.01$, *** $P < 0.001$, **** $P < 0.0001$).
